# Comparing the demographics and laboratory biomarkers of the COVID-19 Omicron wave and the Alpha wave in a predominantly Afro-Caribbean patient population in New York City

**DOI:** 10.1186/s41479-022-00099-w

**Published:** 2022-11-25

**Authors:** Hye Won Shin, Alecia James, Theresa Feng, Lillian Chow, Robert Foronjy

**Affiliations:** 1 College of Medicine, State University of New York, Downstate Health Sciences University, 450 Clarkson Ave, Brooklyn, NY 11203 USA; 2 Department of Epidemiology and Biostatistics, School of Public Health, State University of New York, Downstate Health Sciences University, Brooklyn, NY 11203 USA; 3 Department of Anesthesiology, State University of New York, Downstate Health Sciences University, Brooklyn, NY 11203 USA; 4 Department of Medicine, State University of New York Downstate Health Sciences University, Brooklyn, NY 11203 USA

**Keywords:** COVID-19, Omicron wave, Alpha wave, COVID-19 in African Americans, Laboratory biomarkers, Disease severity

## Abstract

**Background:**

There is a knowledge gap of specific characteristics linked to disease severity of the different COVID-19 waves, especially in underserved populations. We compared the demographic and clinical factors associated with SARS-CoV-2-infected patients admitted to the intensive care unit (ICU) during the Omicron and Alpha waves.

**Methods:**

An observational study comparing two COVID-19 waves was conducted in Brooklyn, NY. Twenty-seven ICU admitted patients with a positive COVID-19 test result during the period of November 1, 2021, to January 31, 2022, (“Omicron wave”) were compared to 271 COVID-19 patients who received ICU consults during the Alpha wave, the period from March 28, 2020, to April 30, 2020.

**Results:**

The Omicron wave had a 55.6% mortality rate compared to a 67.2% mortality rate in the Alpha wave. For the non-survivors, there were more females (66.7%) in the Omicron wave, while the trend was reversed in the Alpha wave (38.5%). Most of the patients seen were Black (> 85%) in both waves. A bivariate comparison of the two waves found that patients in the Omicron wave had overall significantly lower ALT levels (*p* = 0.03) and higher monocyte % (*p* = 0.005) compared to the patients in the Alpha wave. In the multivariate analysis, adjusting for age and sex, increasing levels of HCO3- were significantly associated with reduced mortality in the Omicron wave (OR: 0.698; 95% CI: 0.516 – 0.945; *p* = 0.02). Also, multivariable analyses using both waves combined found that neutrophil % was significantly associated with increased mortality (OR: 1.05; 95% CI: 1.02 – 1.09; *p* = 0.006) while lymphocyte % was significantly associated with reduced mortality (OR: 0.946; 95% CI: 0.904 – 0.990; *p* = 0.018).

**Conclusions:**

The COVID-19-positive ICU patients in the Omicron wave experienced less severe outcomes than those of the Alpha wave. In contrast to the Alpha variant, the Omicron variant exhibited enhanced infectivity and disease severity in females.

## Background

While studies are starting to show that the most recent coronavirus disease 2019 (COVID-19) wave caused by the Omicron variant resulted in a less severe disease compared to other variants [1–5], the Black community continues to be disproportionately burdened by COVID-19 [6–10]. To identify specific disease characteristics and clinical outcomes of the different waves of COVID-19, particularly in the Afro-Caribbean community, this study compared characteristics of SARS-CoV-2-infected patients in the intensive care unit of a single-center hospital in Brooklyn, New York, in two COVID-19 waves. In doing so, we hope to add valuable clinical information regarding the continuously evolving SARS-CoV-2 virus.

## Methods

### Registration

The SUNY Downstate Institutional Review Board approved this study (#1,609,410–1) as minimal‐risk research, which waived the requirement for informed consent.

### Study design and setting

A retrospective, single-center, observational study comparing two COVID-19 waves was conducted at the University Hospital at Downstate (UHD), Brooklyn’s only academic hospital serving its 2.5 million residents. Twenty-seven critical care admitted patients with a positive COVID-19 test result during the period of November 1, 2021, to January 31, 2022, (“Omicron wave”) were compared to 271 COVID-19 patients who received critical care consults during the first wave “Alpha wave,” from March 28, 2020, to April 30, 2020. Data collection for the Alpha wave is described in the Feng et al*.* study [11].

### Participants

Patients were identified by searching UHD’s electronic medical record (HealthBridge, Eclipsys Sunrise) for critical care admitted patients with a positive COVID-19 test result during the time period of interest (November 1, 2021, to January 31, 2022). This period is labeled as the “Omicron wave.” A SARS-CoV-2 infection was confirmed by a positive result on a nasal swab SARS-CoV-2 polymerase chain reaction (PCR) test.

In total, 117 patients were admitted to the Intensive Care Unit (ICU) during the Omicron wave. Out of the 117 patients, only 27 cases tested positive for COVID-19, and these were used in the final analysis. All patients were followed up until they were either discharged from the hospital or expired during hospital admission. If a patient was readmitted during the time period of interest, only the first admission data were used in the final analysis. Patient data on demographics, vitals, comorbidities, and laboratory findings at the time of admission to the ICU were retrieved from the electronic medical record. Also, ICU acceptance status, treatment strategies, COVID vaccination status, and outcomes were obtained. Patients were considered COVID-19-vaccinated if they received the full dosages of the primary vaccine series, which could have been a one- or two-dose series depending on the vaccine brand used.

### Study variables

Variables that were analyzed included age, sex, height, weight, body mass index, vital signs, sodium (Na), bicarbonate (HCO_3_^−^), blood urea nitrogen (BUN), creatinine (Cr), aspartate aminotransferase (AST), alanine transaminase (ALT), total bilirubin, neutrophil percentage (Neu %), lymphocyte percentage (Lym %), monocyte percentage (Mono %), eosinophil percentage (Eos %), basophil percentage (Baso %), platelets (PLT), white blood cell (WBC) count, and hemoglobin. Several variables, such as procalcitonin, C-reactive protein (CRP), and prothrombin time/partial thromboplastin time (PT/PTT), were excluded from the analysis due to inadequate data for these variables. Also, the COVID vaccine status was included for analysis in this study which was confirmed at the time of critical care admission.

### Data sources and measurement

The data in the chart included notes by the critical care provider, other admitting providers, and discharging provider. A COVID-19-related admission to the ICU was classified by a positive SARS-CoV-2 RT-PCR result. Any positive COVID-19 test result 14 or more days before hospitalization and after discharge was considered critical care admission not related to COVID-19. Also, these non-COVID-19-related admissions were confirmed by the admitting provider’s reason for admission, which affirmed that their admissions were not related to a SARS-CoV-2 infection.

### Bias

Selection of subjects for this retrospective study is not based on a specific outcome of interest. Rather, patients meeting eligibility criteria for this study were considered (as described under the “participants” section), which eliminates any potential bias in our subject selection.

### Statistical methods

Several analyses were performed in this comparison study. For one set of analyses, patients in the Omicron wave were stratified by survival status from which demographic, and lab characteristics were obtained.

We further performed separate analyses comparing all the survivors, and all the non-survivors, stratified by wave. Lastly, a comparison was done between all the Alpha wave patients and all the Omicron wave patients. These analyses also yielded demographic and lab characteristics.

For these analyses, Chi-square tests were used to compare categorical variables while independent samples t-tests were used for continuous variables. Categorical variables are expressed as frequencies and percentages, and continuous variables are expressed as means and standard deviations.

Multivariable models were constructed to determine the factors that independently predicted mortality. For the Omicron wave, a model was fitted using HCO_3_^−^ as a predictor (adjusting for age and sex) as this was the only variable that was significant at the bivariate level. Of note, in our previous study using patient data from the Alpha wave [11], we fitted individual models to determine the independent effect of several factors including procalcitonin, CRP, and PLT. A detailed description of that analysis can be found in the literature [11]. Additionally, in the current study, based on factors that were significant in the separate waves (Alpha and Omicron) and data availability, we further fitted individual multivariable models for all patients (both waves combined) using HCO_3_^−^, BUN, Neu %, Lym %, and PLT as predictors of mortality. Models were adjusted for age and sex.

To account for the time that the patients have spent in the study, we performed a survival analysis as this technique can yield valuable clinical information in lieu of a binary indicator of whether an event occurred. For our analyses, the event endpoint is mortality, and the “time to event” is defined as the time taken to reach this endpoint after patient admission. Patients who have not experienced the event from the time of admission have been right censored on the date of their discharge or at the end of the study. The study duration for the survival analysis was 60 days.

The Kaplan Meier method was used to estimate the survival function for the waves of interest (Alpha and Omicron). The survival curves for these waves are based on the outcomes (censored or expired) of admitted patients during these waves. The log rank test was used to estimate if the survival probabilities differed significantly. All analyses were performed using SAS® 9.4 software (SAS Institute Inc., Cary, NC, USA). A *P* < 0.05 (two‐sided) was considered statistically significant.

## Results

### Patient demographics of the Omicron wave

Table 1 shows the demographic characteristics of the Alpha and Omicron waves. The average age of the patients in the Omicron wave was 65.3 $\pm$ 11.4. This wave also included more females (*n* = 16, 59.3%) than males (*n* = 11, 40.7%), and most of the patients were Black/African American (*n* = 23, 85.2%). Also, there were 12 (44.4%) survivors and 15 non-survivors (55.6%) in the Omicron wave. SOFA scores were calculated on 27 subjects using MDcalc (New York, NY, USA). The mean SOFA score at admission of this cohort was 5.63 ± 4.01.

**Table 1:** Characteristics of Alpha and Omicron Waves

Characteristic	All Patients by Wave (*n* = 298)			All Non-Survivors by Wave (*n* = 197)			Omicron Wave by Survival Status (*n* = 27)		
	Alpha (*n*=271)	Omicron (*n* = 27)	*p*-value	Alpha (*n*=182)	Omicron (*n* = 15)	*p*-value	Survivor (*n*= 12)	Non-Survivor (*n* = 15)	*p*-value
	n (%)	n (%)		n (%)	n (%)		n (%)	n (%)	
	271 (90.1%)	27 (9.06%)		182 (92.4%)	15 (7.61%)		12 (44.4%)	15 (55.6%)	0.56
Age (mean, sd.)	66.6 $\pm$ 12.8	65.3 $\pm$ 11.4	0.60	69 $\pm$ 11.4	68.7 $\pm$ 10.4	0.91	61.2 $\pm$11.7	68.7 $\pm 10.4$	0.09
Sex									
Male	157 (57.9)	11 (40.7)	0.08	112 (61.5)	5 (33.3)	0.03	6 (50.0)	5 (33.3)	0.38
Female	114 (42.1)	16 (59.3)		70 (38.5)	10 (66.7)		6 (50.0)	10 (66.7)	
Race									
Black	239 (88.2)	23 (85.2)	0.54	165 (90.7)	15 (100)	0.67	8 (66.7)	15 (100.00)	0.12
White	11 (4.06)	1 (3.70)		7 (3.85)	0 (0)		1 (8.33)	0 (0.00)	
Hispanic	6 (2.21)	0 (0)		2 (1.10)	0 (0)		0 (0.0)	0 (0.0)	
Asian	2 (0.74)	1 (3.70)		0 (0)	0 (0)		1 (8.33)	0 (0.0)	
Unknown	13 (4.80)	2 (7.41)		8 (4.40)	0 (0)		2 (16.7)	0 (0.0)	
Comorbidities									
Asthma	25 (9.23)	5 (18.5)	0.13	17 (9.34)	3 (20.0)	0.19	2 (16.7)	3 (20.0)	0.82
COPD	22 (8.12)	2 (7.41)	0.90	16 (8.79)	1 (6.67)	0.78	1 (8.33)	1 (6.67)	0.87
Diabetes	152 (56.1)	16 (59.3)	0.75	104 (57.1)	9 (60.0)	0.83	7 (58.3)	9 (60.0)	0.93
Hypertension	211 (77.9)	22 (81.5)	0.66	148 (81.3)	12 (80.0)	0.90	10 (83.3)	12 (80.0)	0.82
HIV	8 (2.95)	1 (3.70)	0.83	7 (3.85)	0 (0)	0.44	1 (8.33)	0 (0.00)	0.25
CKD	41 (15.1)	7 (25.9)	0.15	29 (15.9)	5 (33.3)	0.09	2 (16.7)	5 (33.3)	0.33
Covid-19 Vaccination Status									
Yes							4 (33.3)	8 (53.3)	0.15
No							8 (66.7)	5 (33.3)	
Unknown							0 (0)	2 (13.3)	

^*^Columns may not sum to 100 due to rounding

### Comparing the patient demographics of the two waves

The total number of COVID-19-positive critical care patients in the Omicron wave (27 patients) was substantially fewer than that of the first Alpha wave (271 patients) (Table 1). In the Omicron wave, of the 27 COVID-19-positive patients, there was a total of 15 non-survivors, representing a 55.6% mortality rate (Table 1). In comparison, in the Alpha wave [11], of the total 271 critical care patients with positive COVID-19 results, there were 182 non-survivors, representing a 67.2% mortality rate, which was higher than that of the Omicron wave. For the non-survivors, there were more females (*n* = 10, 66.7%) than males (*n* = 5, 33.3%) in the Omicron wave while the opposite was true in the Alpha wave [11] (*n* = 70, 38.5% vs. *n* = 112, 61.5%, respectively). In the Omicron wave, the non-survivors (68.7 ± 10.4) were older than the survivors (61.2 ± 11.7) (Table 1), which was also true of the Alpha wave [11] (69 ± 11.4 vs. 61.6 ± 14.1, respectively). Most of the patients seen were Black/African American in the Omicron wave (*n* = 23, 85.2%) and the Alpha wave [11] (*n* = 239, 88.2%) (Table 1). Also, the majority of the non-survivors in both waves were Black, accounting for 165 (90.7%) non-survivors in the Alpha wave [11] and all the non-survivors in the Omicron wave (*n* = 15, 100%) (Table 1). Of note, the African American community constitutes 91.2% of the community that the UHD serves.

### Bivariate analyses of the Omicron wave

The most common comorbidities in the Omicron wave were hypertension (81.5%) and diabetes (59.3%) (Table 1), which were similar to the results of the Alpha wave [11]. At the bivariate level, only a decrease in HCO_3_^−^ was significantly associated with mortality (*p* = 0.006) in the Omicron wave (Table 2). Interestingly, in the Omicron wave, the COVID-19 vaccination status results revealed that more non-survivors (*n* = 8, 53.3%) were vaccinated than survivors (*n* = 4, 33.3%) (Table 1).

**Table 2:** Laboratory Findings of Alpha and Omicron Waves

Factors	All Patients by Wave (*n* = 298)					All Non-Survivors by Wave (*n* = 197)					Omicron Wave by Survival Status (*n* = 27)				
		Alpha (*n* = 271)		Omicron (*n* = 27)	*p*- value		Alpha (*n* = 182)		Omicron (*n* = 15)	*p*- value		Survivor (*n* = 12)		Non-Survivor (*n* = 15)	*p*- value
	n	Mean (sd.)	n	Mean (sd.)		n	Mean (sd.)	n	Mean (sd.)		n	Mean (sd.)	n	Mean (sd.)	
Na	270	138.6 $\pm$ 10.0	26	140.7 $\pm$ 6.43	0.14	182	139.4 ± 9.80	15	142.3 ± 7.44	0.17	11	138.5 $\pm$ 4.08	15	142.3 $\pm$ 7.44	0.14
HCO_3_^−^	268	21.0 $\pm$ 6.38	26	21.5 $\pm$ 4.79	0.64	180	20.6 ± 5.95	15	19.4 ± 4.84	0.39	11	24.4 $\pm$ 3.01	15	19.4 $\pm$ 4.84	0.006
BUN	270	44.0 $\pm$ 41.2	26	54.0 $\pm$ 36.4	0.20	182	47.9 ± 45.1	15	58.5 ± 29.1	0.21	11	47.9 $\pm$ 45.3	15	58.5 $\pm$ 29.1	0.48
Cr	270	2.76 $\pm$ 3.14	26	3.20 $\pm$ 2.79	0.45	182	2.91 ± 3.27	15	3.26 ± 2.41	0.60	11	3.10 $\pm$ 3.36	15	3.26 $\pm$ 2.41	0.88
AST	249	120.0 $\pm$ 340.4	25	100.4 $\pm$ 161.0	0.62	164	139.4 ± 404.1	14	100.9 ± 166.1	0.48	11	99.7 $\pm$ 162.2	14	100.9 $\pm$ 166.1	0.99
ALT	249	67.4 $\pm$ 137.4	25	40.2 $\pm$ 44.4	0.03	164	74.4 ± 160.5	14	46.5 ± 43.1	0.11	11	32.3 $\pm$ 46.8	14	46.5 $\pm$ 43.1	0.44
Total Bilirubin	250	0.840 $\pm$ 1.08	25	1.22 $\pm$ 2.83	0.51	165	0.866 ± 1.15	14	1.54 ± 3.77	0.52	11	0.827 $\pm$ 0.706	14	1.54 $\pm$ 3.77	0.55
Neu %	221	82.6 $\pm$ 8.42	25	77.6 $\pm$ 13.7	0.09	151	83.9 ± 7.48	13	79.7 ± 13.6	0.29	12	75.3 $\pm$ 14.1	13	79.7 $\pm$ 13.6	0.44
Lym %	227	9.94 $\pm$ 6.69	24	13.8 $\pm$ 11.8	0.13	154	9.06 ± 5.62	13	12.2 ± 12.4	0.38	11	15.7 $\pm$ 11.4	13	12.2 $\pm$ 12.4	0.48
Mono %	227	4.40 $\pm$ 2.23	24	6.41 $\pm$ 3.11	0.005	154	4.35 ± 2.24	13	5.98 ± 3.36	0.11	11	6.91 $\pm$ 2.85	13	5.98 $\pm$ 3.36	0.48
Eos %	214	0.526 $\pm$ 0.892	24	0.483 $\pm$ 0.925	0.83	147	0.412 ± 0.572	13	0.162 ± 0.236	0.005	11	0.864 $\pm$ 1.27	13	0.162 $\pm$ 0.236	0.06
Baso %	210	0.418 $\pm$ 0.327	24	0.450 $\pm$ 0.582	0.79	144	0.406 ± 0.307	13	0.469 ± 0.641	0.73	11	0.427 $\pm$ 0.535	13	0.469 $\pm$ 0.641	0.86
PLT	263	249.0 $\pm$ 123.7	26	220.4 $\pm$ 93.7	0.16	178	228.4 ± 111.7	14	237.7 ± 116.8	0.78	12	200.3 $\pm$ 54.8	14	237.7 $\pm$ 116.8	0.32
WBC	264	11.7 $\pm$ 5.70	26	10.1 $\pm$ 7.19	0.28	179	11.7 ± 5.70	14	10.7 ± 8.25	0.66	12	9.26 $\pm$ 5.96	14	10.7 $\pm$ 8.25	0.61
Hemoglobin	264	12.2 $\pm$ 2.43	26	11.4 $\pm$ 2.51	0.14	179	12.3 ± 2.43	14	11.7 ± 2.75	0.42	12	11.1 $\pm$ 2.27	14	11.7 $\pm$ 2.75	0.53

^*^Columns may not sum to 100 due to rounding

### Comparing the laboratory biomarkers of the two waves

**Fig. 1:**
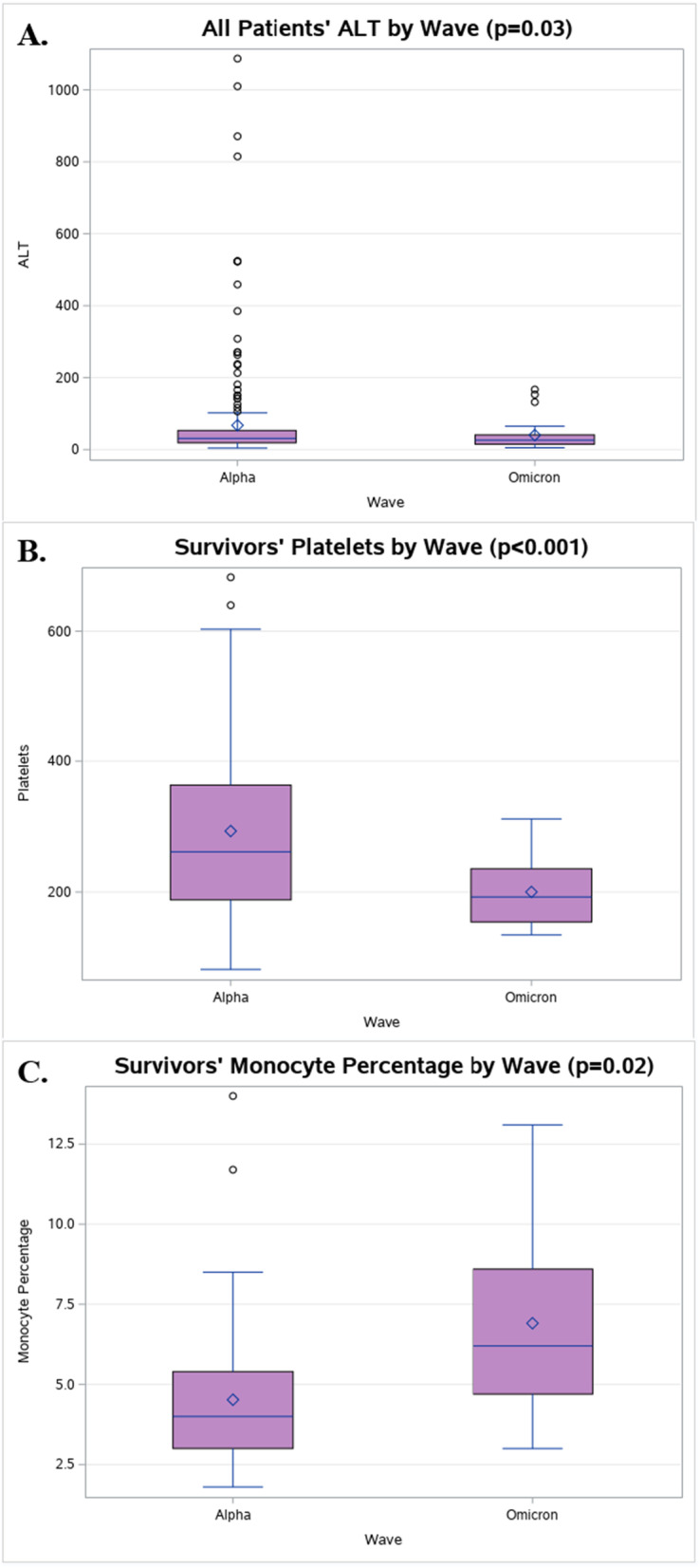
Box-and-whisker plots for laboratory biomarkers by wave. **A** All Patients’ ALT Plot. **B** Survivors’ Platelets Plot. **C** Survivors’ Monocyte % Plot

A comparison of the lab findings of the two waves (shown in Table 2) found that patients in the Omicron wave had overall significantly lower ALT levels (40.2 ± 44.4 vs. 67.4 ± 137.4, *p* = 0.03) (Fig. 1A) and higher Mono % (6.41 ± 3.11 vs. 4.40 ± 2.23, *p* = 0.005) compared to the patients in the Alpha wave. Also, as shown in Table 2, the non-survivors in the Omicron wave had significantly lower Eos % (0.162 ± 0.236 vs. 0.412 ± 0.572, *p* = 0.005) compared to the non-survivors in the Alpha wave. Table 3 shows that the survivors in the Omicron wave had significantly lower PLT (200.3 ± 54.8 vs. 292.2 ± 136.7, *p* =  < 0.001) (Fig. 1B) and higher Mono % (6.91 ± 2.85 vs. 4.52 ± 2.24, *p* = 0.02) (Fig. 1C) compared to the survivors in the Alpha wave. In addition, in the Alpha wave [11], 38.7% (*n* = 105) of the patients required invasive mechanical ventilation while in the Omicron wave, 29.6% (*n* = 8) of the patients required invasive mechanical ventilation at the time of ICU admission.

**Table 3:** Laboratory Findings of All Survivors by Wave

Factors	All Survivors by Wave (*n* = 101)				
		**Alpha (*n* = 89)**		**Omicron (*n* = 12)**	***p*- value**
	n	Mean (sd.)	n	Mean (sd.)	
Na	88	137.1 $\pm$ 10.3	11	138.5 $\pm$ 4.08	0.38
HCO_3_^−^	88	22.0 $\pm$ 7.11	11	24.4 $\pm$ 3.01	0.05
BUN	88	36.0 $\pm$ 30.7	11	47.9 $\pm$ 45.3	0.42
Cr	88	2.44 $\pm$ 2.85	11	3.10 $\pm$ 3.36	0.54
AST	85	82.4 $\pm$ 151.5	11	99.7 $\pm$ 162.2	0.74
ALT	85	54.0 $\pm$ 73.6	11	32.3 $\pm$ 46.8	0.20
Total Bilirubin	85	0.791 $\pm$ 0.931	11	0.827 $\pm$ 0.706	0.88
Neu %	70	79.9 $\pm$ 9.66	12	75.3 $\pm$ 14.1	0.30
Lym %	73	11.8 $\pm$ 8.25	11	15.7 $\pm$ 11.4	0.29
Mono %	73	4.52 $\pm$ 2.24	11	6.91 $\pm$ 2.85	0.02
Eos %	67	0.776 $\pm$ 1.32	11	0.864 $\pm$ 1.27	0.84
Baso %	66	0.442 $\pm$ 0.368	11	0.427 $\pm$ 0.535	0.93
PLT	85	292.2 $\pm$ 136.7	12	200.3 $\pm$ 54.8	< 0.001
WBC	85	11.5 $\pm$ 5.73	12	9.26 $\pm$ 5.96	0.25
Hemoglobin	85	11.9 $\pm$ 2.41	12	11.1 $\pm$ 2.27	0.26

^*^Columns may not sum to 100 due to rounding

### Multivariable analyses

Table 4 shows the results of the multivariable model with HCO_3_^−^ as a predictor of mortality in the Omicron wave. Adjusting for age and sex, increasing levels of HCO_3_^−^ were significantly associated with a 30% reduced odds of mortality (OR: 0.698; 95% CI: 0.516 – 0.945; *p* = 0.02). The results of the multivariable analyses using both waves combined are shown in Table 5. Significant associations were found for neutrophils where increasing neutrophil percentage was associated with a 5% increased odds of mortality (OR: 1.05; 95% CI: 1.02 – 1.09; *p* = 0.006). Also, increasing lymphocyte percentage was independently associated with a 5% reduced likelihood of mortality (OR: 0.946; 95% CI: 0.904 – 0.990; *p* = 0.018).

**Table 4:** Logistic Regression Model for Bicarbonate Predicting Mortality in the Omicron Wave

Factors	OR (95% CI)	*P* value
HCO_3_^−^	0.698 (0.516 – 0.945)	0.02
Age	1.13 (0.995 – 1.29)	0.06
Sex (Referent = Male)	0.688 (0.06 – 7.76)	0.76

**Table 5:** Logistic Regression Models for Factors Predicting Mortality in All Patients (Alpha and Omicron Wave combined)

Factors	OR (95% CI)	*P* value
HCO_3_^−^	0.964 (0.925 – 1.01)	0.09
BUN	1.01 (0.998 – 1.02)	0.12
Neut %	1.05 (1.02 – 1.09)	0.006
Lym %	0.946 (0.904 – 0.990)	0.018
PLT	0.997 (0.994 – 0.999)	0.002

^*^Individual models were created for the variables shown, adjusting for age and sex

**Fig. 2:**
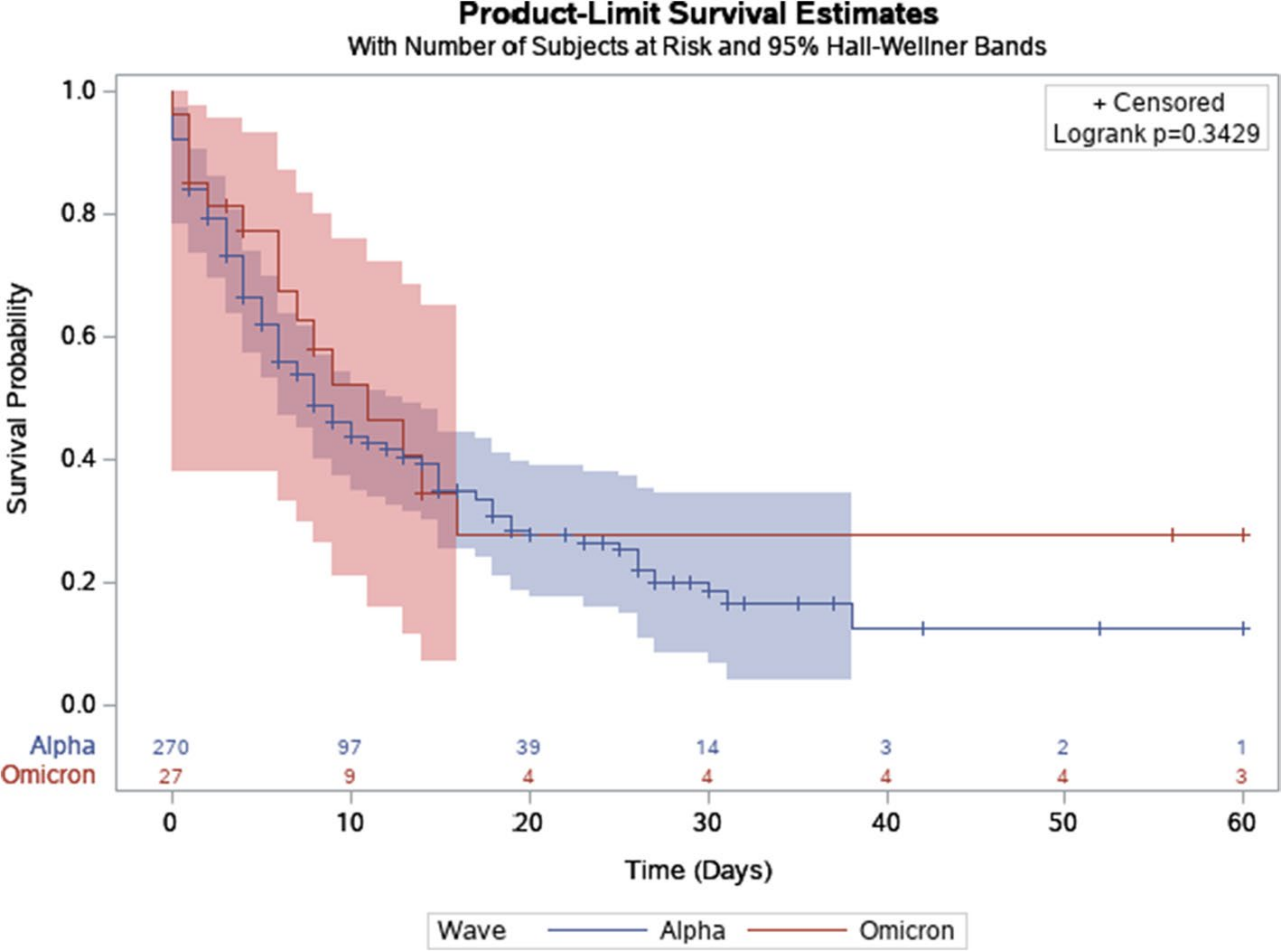
Kaplan Meier curves of patients in the Alpha and Omicron waves

The survival curves for the Alpha and Omicron waves are shown in Fig. 2. The median survival time of patients in the Alpha wave was 8 days while that of the Omicron wave was 11 days; a log rank test, however, found that there was no significant difference in the survival probabilities of patients in the two waves (*p* = 0.34)

## Discussion

This study compared the clinical characteristics and laboratory biomarkers in COVID-19 patients with critical care admissions during the Alpha wave of the COVID-19 pandemic (March 28, 2020, to April 30, 2020) and the Omicron wave (November 1, 2021, to January 31, 2022) at the UHD. Patients in the Omicron wave had differing characteristics and outcomes compared to those in the Alpha wave. The patients admitted to the ICU in the Omicron wave were slightly younger, and there was a higher proportion of females. This finding contrasts with the Alpha wave where men were disproportionately affected and had an increased risk of mortality. In our study of the Omicron wave, these mortality trends were reversed with women constituting the majority of the Omicron related deaths. Recently, a large review of COVID19 outcomes identified a higher prevalence of Omicron infection amongst females [10]. While the mortality of the Omicron variant is significantly lower than the Alpha variant, the increased prevalence of Omicron infection in females could have long-term health consequences as women have an increased risk of developing long haul COVID [12]. It is conceivable that gender related differences in cell mediated immunity or changes in the spike protein in the Omicron variant could skew infection towards females. However, further research is needed to better understand why these variants exhibited varying sex predilections.

Compared to the Alpha wave, the Omicron wave had much fewer ICU admissions (27 vs. 271) and a lower mortality rate (55.6% vs. 67.2%), suggesting a decreased disease severity during the Omicron wave. This finding is consistent with studies from South Africa [13–15] and European countries [16, 17]. Also, the mean SOFA score of the Omicron wave was lower than that of the Alpha wave (5.63 ± 4.01 vs. 8.33 ± 0.21) [11]. The lower mortality rate, ICU admissions, and mean SOFA score in the Omicron wave suggest that the Omicron variant, the predominant variant during this period, is associated with less disease severity and mortality compared to the previous SARS-CoV-2 variants as supported by other studies [2–5]. Interestingly, as seen in the Kaplan Meier survival curves (Fig. 2), although there was no significant difference in the survival probabilities of patients in the two waves (*p* = 0.34), the patients in the Alpha wave had longer hospital admission times. Another notable point is that treatments for COVID-19-related hospitalized patients have improved with more research and clinical findings and hospitals overall have more knowledge on the SARS-CoV-2 virus [18, 19], so these advances have also contributed to the overall lower mortality of COVID-19 patients. Lastly, the decreased volume of patients in the Omicron wave placed less strain on ICU resources which may also partly explain the improved outcomes in this cohort.

The UHD serves a predominantly Afro-Caribbean patient population with African Americans representing 91.2% of the local population. Accordingly, most of the patients seen were Black/African American in the Omicron wave (*n* = 23, 85.2%) and the Alpha wave (*n* = 239, 88.2%). Our findings, which showed that the majority of the non-survivors were Black in both waves with 165 patients (90.7%) in the Alpha wave and all of the patients in the Omicron wave (*n* = 15, 100%), could therefore be correlated with our hospital census. Though these numbers are consistent with our local demographics, it appears that the Black population continues to be disproportionately affected by COVID-19. In fact, as of April 2022, Kings County, the county where the UHD is located, continues to be the county with the highest number of COVID-related deaths in the state of New York and the fourth-highest number of deaths nationwide [6]. The high mortality rate in the Black population could be due to the “longstanding inequalities rooted in systemic and pervasive problems in the United States,” [20] as articulated by some scholars. Some examples include an overrepresentation of racial and ethnic minority populations in essential jobs, leading to greater chances of being exposed to COVID-19, or these groups facing multiple barriers to accessing health care [21]. Also, comorbidities, such as hypertension, or obesity, are prevalent in the Black population, thus contributing to worsened disease outcomes [7–9, 21–24]. Addressing the underlying health issues of obesity, diabetes and hypertension in the African American community could be an effective means of reducing the susceptibility of this community to future pandemics.

Mechanical ventilation is an indicative measure of poor disease outcome [25]. All the patients who were admitted to the ICU in the Alpha wave had severe respiratory illness requiring high-level oxygen supplementation or some form of mechanical ventilation [11]; 38.7% (*n* = 105) of the patients required invasive mechanical ventilation at the time of their initial critical care consult. In the Omicron wave, 29.6% (*n* = 8) of the patients required invasive mechanical ventilation. Our finding that there was less invasive mechanical ventilator use in the Omicron wave compared to the Alpha wave is consistent with findings in a study by Modes et al. [26] We also found that while most of the initial consult diagnoses in the Alpha wave were related to a severe respiratory illness, the initial admitting diagnoses in the Omicron wave had many other diagnoses that were not related to a respiratory-related illness. These included diabetic ketoacidosis, non-infectious diarrhea, facial weakness, hypokalemia, and altered mental status. This difference may be because the Omicron variant results in a decreased disease severity possibly due to its lower efficiency of replication in the lung parenchyma [27], which reduces severe respiratory illnesses and the need for invasive mechanical ventilators as observed in the Omicron wave. Indeed, almost fifty percent of the Omicron related deaths occurred in individuals who did not require mechanical ventilatory support. This suggests that infection with the Omicron variant was not the primary factor causing death. Nevertheless, we found that the Omicron variant infection can still cause severe lower respiratory illness as seen in other studies [26].

A bivariate comparison of the laboratory findings of the two waves found that the patients in the Omicron wave had overall significantly lower ALT levels compared to the patients in the Alpha wave. Also, multivariable analyses using both waves found that Neu % was significantly associated with a 5% increased odds of mortality and Lym % was significantly associated with 5% reduced likelihood of mortality. Several studies found that an increased neutrophil ratio, a lower Lym %, and an elevated ALT are independently correlated with disease mortality [11, 28–31]. Accordingly, these parameters support our observation that patients in the Omicron wave experienced a less severe disease, possibly due to less systemic inflammation, better immunological response to viral infection [32], and less liver injury [29]. However, another finding in our study is that the patients in the Omicron wave had a significantly lower PLT count than the patients in the Alpha wave, which conflicts with what would be expected as studies show that thrombocytopenia is associated with a higher risk of poor outcomes [28–30]. This conflicting result may be because a PLT count is reported to have a worse prognostic value than other biomarkers, such as CRP [33]. Also, we found that the Omicron wave patients had a significantly higher Mono % than the Alpha wave patients and higher Mono % in the Omicron wave survivors than Alpha wave survivors. Several studies describe an increased monocyte count in severe COVID-19 disease [34–36], but some studies have shown decreased monocytes in patients with severe disease [37–39]. The contrasting findings in these studies suggest the need for further studies or may suggest that Mono % is not a reliable marker of disease severity due to its dynamic effects.

One factor in the Omicron wave that differs from the Alpha wave was the addition of the COVID-19 vaccine, as the COVID-19 vaccine was developed after the Alpha wave [40]. Many studies show that the vaccine reduces COVID-19-related hospitalization and death [41–43]. Specifically, in regards to critical care, Thompson et al. found that the effectiveness of mRNA-based vaccines was 90% against infection leading to ICU admission [44]. In addition, a recent study conducted by the Centers for Disease Control and Prevention found that there were more hospitalizations during the Omicron-dominant period compared to the Delta-dominant period, but vaccination, including a booster dose, was associated with a lower likelihood of ICU admission [26]. In our study, interestingly, more non-survivors were vaccinated compared to survivors (53.3% vs. 33.3%) during the Omicron wave. The high rate of breakthrough cases and mortality among the vaccinated patients could have a manifold explanation. Firstly, the non-survivors were older than the survivors (mean age = 68.7 vs. 61.2), so the higher age could have been a cofactor in mortality. Secondly, only two patients in the Omicron wave were fully vaccinated with a booster dose, both of whom were non-survivors. Lastly, many of the patients admitted to the ICU have comorbidities, which are particularly more likely in the Black community [7–9, 21–24], and could have also contributed to the higher mortality seen in the vaccinated patients.

On a broader level, our findings reflect a lessening of the overall disease burden of COVID-19 in the Omicron wave, which is indicative of a less-lethal variant, and possibly a higher prevalence of natural immunity amongst the local community. The progress thus far on COVID-19 has been promising, and continued research in this domain is needed, which will be instrumental in addressing COVID-related health disparities and improving clinical outcomes.

### Limitations

The findings in this study should be interpreted in light of several limitations. Firstly, the sample size of the Omicron wave was smaller than the Alpha wave, limiting some statistical analyses and possibly affecting our conclusions. Also, we did not have the sequencing data for each patient to identify the SARS-CoV-2 variant. Nevertheless, the New York City Department of Health reported that nearly all COVID-19 cases were the Omicron variant during the time period studied. In addition, our study population consisted largely of Afro-Caribbeans, so future studies on other patient populations are needed to extrapolate our findings to other racial and ethnic groups. Furthermore, the two waves had different durations, which could have also potentially affected our data. Moreover, patients admitted to ICU require critical care and generally have more severe manifestations compared to other hospitalized patients, limiting the ability to generalize our findings to less severe patient populations. Despite these limitations, to our knowledge, this is the first study to compare the clinical outcomes of COVID-19 critical care patients (of a predominantly Afro-Caribbean community) in the specific waves studied, in Brooklyn, New York. These findings contribute to the growing literature on COVID-19, as different variants emerge, and highlight the outcomes of a generally underserved population, which is vital to their clinical care, and more broadly, informative for developing effective public health interventions.

## Conclusion

In summary, there were fewer COVID-19-positive patients admitted to the ICU and a lower mortality rate in the Omicron wave compared to the Alpha wave, suggesting a reduced disease severity. The reduced disease severity in the Omicron wave may be attributed to the emerging novel therapies and wide-spread vaccination efforts. Nevertheless, COVID-19-positive patients during the Omicron wave still experienced substantial morbidity and mortality. In contrast to the Alpha variant, our findings indicate that the Omicron variant exhibited an enhanced predilection for infection in females. The reasons for these variant related changes in gender susceptibility will require further study.

## Data Availability

The data generated and analyzed during this study are included in this literature.

## Abbreviations

ALT: Alanine transaminase
AST: Aspartate aminotransferase
Baso %: Basophil percentage
BUN: Blood urea nitrogen
COVID-19: Coronavirus disease 2019
Cr: Creatinine
CRP: C-reactive protein
Eos %: Eosinophil percentage
HCO_3_^−^: Bicarbonate
ICU: Intensive care unit
Lym %: Lymphocyte percentage
Mono %: Monocyte percentage
Na: Sodium
Neu %: Neutrophil %
PCR: Polymerase chain reaction
PLT: Platelet
PT/PTT: Prothrombin time/partial thromboplastin time
WBC: White blood cell
